# Levofloxacin-induced rhabdomyolysis: a case report

**DOI:** 10.1186/s13256-016-1004-6

**Published:** 2016-08-24

**Authors:** Febin John, Ruby Oluronbi, C. S. Pitchumoni

**Affiliations:** 1 Department of Internal Medicine, Saint Peters University Hospital, Rutgers-RWJ Medical School, New Brunswick, NJ USA; 2 Department of Internal Medicine, Drexel University College of Medicine, Philadelphia, PA USA

**Keywords:** Rhabdomyolysis, Levofloxacin, Drug-induced rhabdomyolysis, Case report

## Abstract

**Background:**

Rhabdomyolysis secondary to quinolones is not frequent. There are scarce reports in the literature associating rhabdomyolysis to levofloxacin. We describe a case of levofloxacin-induced rhabdomyolysis.

**Case presentation:**

A 52-year-old African-American man presented with muscle tightness after taking three doses of levofloxacin. He had elevated creatine kinase without acute kidney injury. His symptoms resolved after discontinuation of levofloxacin and supportive care.

**Conclusions:**

It is fascinating that our patient has a prior history of rhabdomyolysis, likely from levofloxacin. Our case highlights the need to be mindful of this potentially life-threatening complication of levofloxacin.

## Background

Levofloxacin is a deoxyribonucleic acid (DNA) gyrase inhibitor that inhibits the relaxation of supercoiled DNA, thus promoting DNA strand breakage. Well-known adverse reactions of levofloxacin are gastrointestinal (nausea and vomiting approximately 5 %), central nervous system (CNS; seizure <1 %), cardiovascular (arrhythmia <1 %), and musculoskeletal (tendinitis <1 %) [1]. We describe a less-known side effect of levofloxacin, which is a commonly prescribed antibiotic in the United States of America (USA) [2].

## Case presentation

A 52-year-old African-American man with beta thalassemia, gout, and hypertension presented with the chief complaint of tightness and fatigue in his thigh muscles for 2 days that subsequently involved his shoulders and arms causing difficulty with ambulation. He did not have any prodromal or provoking events except that he took three doses of levofloxacin 750 mg prescribed for a lower respiratory tract infection prior to the onset of his symptoms. By the third dose, along with the worsening of symptoms he noticed discoloration of urine. He had a history of rhabdomyolysis 4 years earlier thought to be secondary to levofloxacin. His vital signs were normal and a physical examination was significant for generalized muscle tenderness. His urine was dark brown and turbid, and negative for glucose, bilirubin, ketones, leukoesterase, and nitrite but with 4+ blood and 0 to 2 red blood cells per highpower field. Other laboratory results during the current episode along with a comparison to the prior episode are summarized in Table 1.

**Table 1:** Comparison of laboratory values during two episodes of rhabdomyolysis

Laboratory test	2015	2011	Normal range
White blood cell count	11.9	7.3	4.0–11.0 cells per μL
Hemoglobin	11.1	9.7	13.0–17.0 g/dL
Hematocrit	33.3	29.8	40.0–56.0 %
Mean corpuscular volume	60.6	66.6	80.0–100.0 fL
Red cell count	5.49	4.47	4.40-6.20 106/mm^3^
Red cell distribution width	22.5	23.3	11.0–16.0 %
Blood urea nitrogen	18	17	6–20 mg/dL
Serum creatinine	0.95	0.77	0.66–1.25 mg/dL
Serum sodium	136	138	136-145 mmol/L
Serum potassium	4.2	4.2	3.5-5.1 mmol/L
Serum phosphorus	3.6	3.3	2.7-4.5 mg/dL
Thyroid-stimulating hormone	3.4	2.72	0.465-4.68 μIU/mL
Aspartate aminotransferase (AST)	445	1137	17–59 U/L
Alanine aminotransferase (ALT)	133	314	21–72 U/L
Alkaline phosphatase (ALP)	50	78	56–119 U/L
Creatine kinase (CK)	92,950	268,666	55–170 U/L
Creatine kinase MB fraction	9.2	6.4	0.6–6.3 ng/mL
Lactate dehydrogenase	5542	21500	140–271 U/L
*Mycoplasma pneumoniae* antibody IGM	Negative	–	
Influenza A and B rapid antigen	Negative	Negative	

IgM, Immunoglobulin M; Cells per μL, cells per micro liter; g/dL, gram per deci liter; fL, femto liter; 10^6^/mm3, 10^6^ per cubic millimeter; mmol/L, millimol per liter; μIU/mL, micro international units per milliliter; U/L, units per liter; ng/mL, nano gram per milliliter

Levofloxacin was discontinued and aggressive intravenous hydration was started. His creatine kinase (CK) gradually dropped over the next 10 days to <5000 U/L. His serum creatinine remained <1 mg/dL. His aspartate aminotransferase (AST) peaked at 1298 U/L while alanine aminotransferase (ALT) remained around 300 U/L and both tapered to a normal level. His clinical improvement correlated with the drop in CK.

## Discussion

Rhabdomyolysis is a severe form of necrotizing myopathy that occurs after acute muscle insult. Drug-induced rhabdomyolysis is well documented and can occur via primary or secondary myotoxic effect. Imbalance between production and utilization of adenosine triphosphate (ATP) in the muscle caused by drugs can lead to direct toxic effect on the sarcolemma. Secondary myotoxicity can occur via CNS depression causing prolonged immobilization and pressure on large muscles [3]. Common causes of rhabdomyolysis such as trauma, exertion, or seizure were absent in this case. Hypophosphatemia, hypokalemia, hypothyroidism, and infections (influenza, mycoplasma) were excluded in our patient. His medications were reviewed and none of them were known to have this adverse reaction [4]. The fact that he recovered after discontinuation of the drug implies the contributory role of levofloxacin.

The earliest report of quinolone-induced rhabdomyolysis was observed by Blain *et al*. in 1996 [1]. Levofloxacin-induced rhabdomyolysis was first reported in 2000 and thus far only a few cases have been reported [4–7]. Previous reports that rhabdomyolysis occurs within 6 days of initiation of levofloxacin remained true in our case also. The exact mechanism by which levofloxacin induces rhabdomyolysis is not known yet. A theory of vascular hyperpermeability is hypothesized. Localized edema and mononuclear cells in muscles of animal models after a systemic dose of fluoroquinolones support this hypothesis. Tumor necrosis factor α (TNFα), interleukin 1 (IL-1), histamine, and plasma fibronectin are some of the mediators involved in this vascular hyperpermeability [4]. Levofloxacin can also lead to seizure activity and muscle breakdown by lowering the seizure threshold; however, there was no seizure activity in our patient.

Liver enzyme elevation, as seen in our patient, is common in rhabdomyolysis. ALT, a cytosolic enzyme is found mostly in the liver. On the other hand, AST, which exists in two isoforms (cytosolic and mitochondrial), is found in the liver, heart, skeletal muscles, kidney, brain, and pancreas. Elevated AST is seen in approximately 93 % of cases and the normalization parallels the fall in CK levels [8]. However, only 75 % of cases of rhabdomyolysis have ALT elevations and its fall does not correlate with CK level. Elevations of liver enzymes in rhabdomyolysis may not be indicative of liver injury [8].

Diagnosis of rhabdomyolysis is by elevations in CK in the blood. Although myoglobinuria can occur in 57 % of patients, it often resolves before the rise in CK. Hence, the absence of myoglobinuria does not rule out the diagnosis [3, 4]. Management is by stopping the offending agent along with adequate hydration to prevent acute renal failure. Systemic steroids although reported to have a beneficial effect, were not used in this case due to lack of scientific evidence [7].

## Conclusions

Our case is fascinating given the prior history of rhabdomyolysis establishing a cause–effect relationship of levofloxacin in the etiology of rhabdomyolysis. Thus it is important to be aware and suspect this infrequent but fatal side effect of this commonly prescribed antibiotic.

## Abbreviations

ALT, alanine aminotransferase; AST, aspartate aminotransferase; ATP, adenosine triphosphate; CK, creatine kinase; CNS, central nervous system; IL-1, interleukin 1; TNFα, tumor necrosis factor α
